# Editorial: Challenges for the provision of guideline-recommended cancer care to rural and medically underserved communities

**DOI:** 10.3389/frhs.2022.1113916

**Published:** 2023-01-10

**Authors:** Reza Yousefi Nooraie, Katia Noyes

**Affiliations:** ^1^ Department of Public Health Sciences, School of Medicine and Dentistry, University of Rochester, Rochester, NY, United States; ^2^ Division of Health Services and Policy, Department of Epidemiology and Environmental Health, School of Public Health and Health Professions, University at Buffalo, Buffalo, NY, United States

**Keywords:** cancer care, barriers to care, implementation, disparities (health), financial toxicities, sustainability

**Editorial on the Research Topic** Challenges for the provision of guideline-recommended cancer care to rural and medically underserved communities

Health disparities have persisted in cancer care despite extensive research and a decades-long mandate to eliminate them (1). Many leading research and policy organizations have specifically identified rural cancer disparities as one of the important priorities for public health and social justice (2–4). While a number of international studies have identified factors contributing to the disparities in cancer care and proposing programs and demonstrations to address the problem, only a few healthcare organizations in a hand-full of countries have been able to successfully implement and scale up such programs (4–8).

The topic objective is to reveal some of the hidden underlying causes of unequal access to cancer care and develop tailored interventions and strategies to mitigate these barriers. In essence, the topic bridges across the disciplinary fields of health services research, dissemination & implementation science, health equity, policy, economics and behavioral research and includes diverse issues such as health insurance, workforce shortages, financial toxicity (FT), patient preferences and inequities in health care access. The gap between what is known to optimize healthcare delivery and what is actually implemented in everyday practice remains one of the most important issues hindering the healthcare systems and public health around the world. Finding ways to enhance access and awareness of patients, providers and healthcare organizations (dissemination) and to facilitate adoption and integration of best evidence into practice (implementation) are essential to improving health care and health outcomes in underserved communities.

Healthcare organizations and agencies in the public and private sectors spend billions of dollars on research and service delivery programs each year, yet patients and stakeholders often lack sufficient information to make decisions regarding the most effective treatment strategies for their particular condition that is available to them locally. One of the key reasons why evidence-based interventions often do not deliver the expected benefits in community settings is implementation failure (9–11). Restricted staffing, technology, financing and lack of strategic leadership in community oncology clinics pose significant challenges for adaptation, implementation and dissemination of evidence-based practices in such settings (12–14).

**Figure 1:**
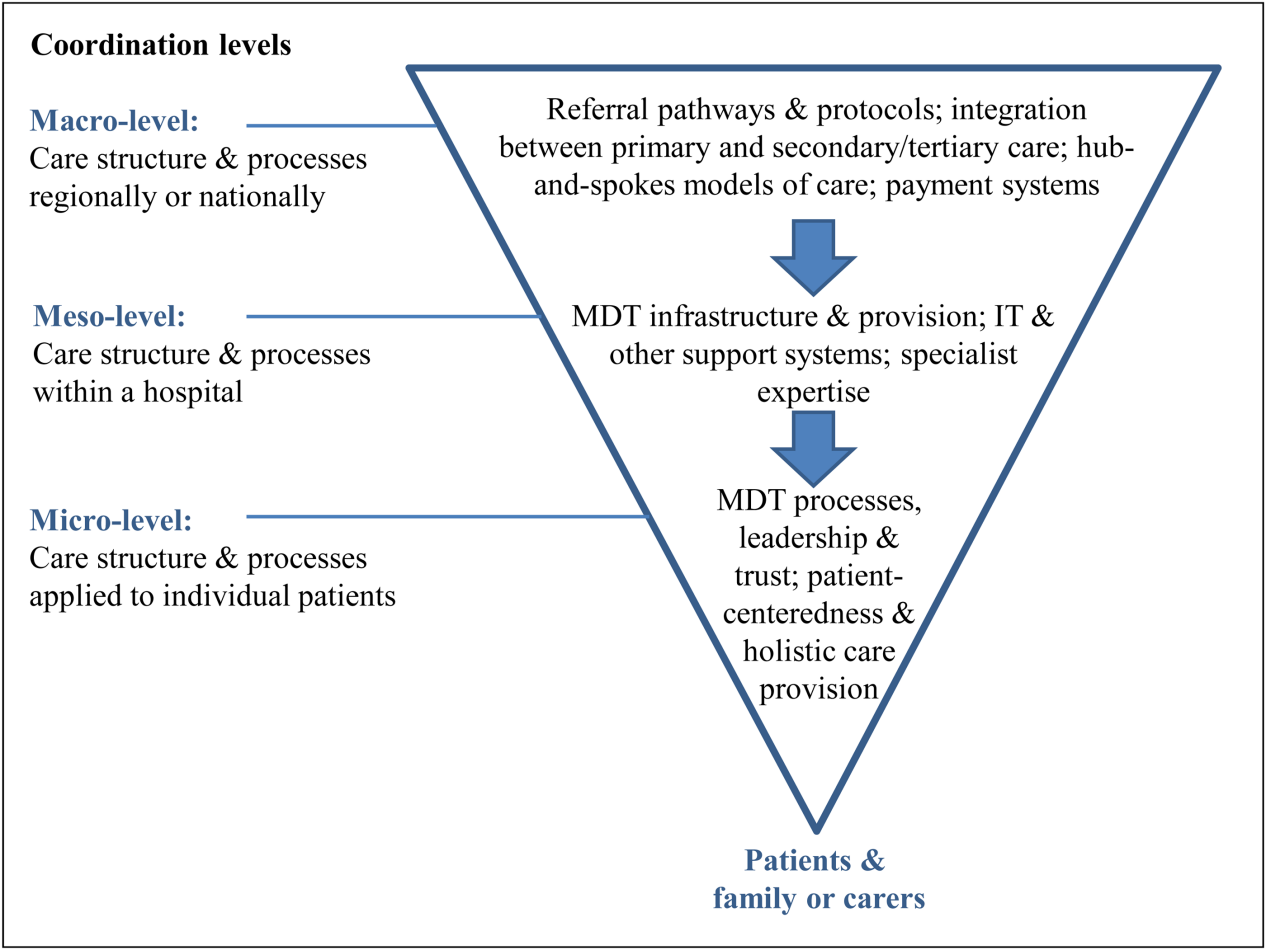
Levels of implementation strategies required for cancer care delivery—from a regional care system to the care of individual patients (15). Adopted from Noyes et al. (2016). MDT, multi-disciplinary teams.

Dissemination, implementation, and sustainment of best practices and evidence-based interventions require supportive infrastructures and coordination among various levels of healthcare system, spanning from regional care systems to individual point of care (Figure 1).

The studies in this series provided insight into the complexity of implementation barriers and strategies to facilitate it at various levels of healthcare systems. They also addressed the complexity of implementation at various stages of implementation journey, spanning from pre-implementation planning to post-implementation evaluation.

Crabtree-Ide et al. assessed barriers to implement guideline-concordant cancer services in rural communities, at pre-implementation stage, and proposed potential implementation strategies to improve implementation at regional level. Team-based cancer care delivery and development of regional networks were identified as the most pressing strategies. While multidisciplinary cancer care teams have become a norm at large academic cancer centers, rural and small free-standing community oncology clinics rarely possess the resources and personnel to form multidisciplinary teams within and across the regions they serve (Crabtree-Ide et al.).

Lack of capacity (time, expertise and reimbursement) among community oncology clinics and rural hospitals to offer guideline-recommended cancer interventions has been identified as the main barrier to successful implementation of skills training in community settings, where more than 80% of US adult cancer patients receive care (16, 17). In their mixed-methods, interventional study of implementing an evidence-based intervention to motivate healthcare professional implement a smoking cessation counselling service, De Frel et al. have demonstrated that without thoughtful planning and decision support, many overextended and burned out hospital providers do not have time even for simple intervention like assessing patient smoking status.

Lack of patient transportation is one of the most common logistical barriers to cancer screening and access of cancer supportive services. While the solutions to this problem, like rideshares, are wide available, their integration with healthcare system is not without challenges as demonstrated by Bell-Brown et al. Scheduling non-emergency medical transportation (NEMT) services requires infrastructure and coordination at various levels. One important requirement is a Health Insurance Portability and Accountability Act (HIPAA) compliant platform, that would allow to bill the costs of transportation directly to health insurance organization, and ensures safety of patients after sedation (e.g., endoscopy).

Wahlen et al. presented the first study that pro-actively and systematically planned for evidence-based adaption of a successful regional cancer care network for the needs and capacity of rural hospitals. To address the complexities of implementation, they distilled the intervention into its core functions that could be optimized and assessed in future interventional studies.

Individuals historically underserved by medical institutions (e.g., patients living in rural areas, non-English speaking patients, patients of color) experience disproportionate financial burden and poor access to cancer care (22). For cancer care providers to more effectively reach and assist patients with disproportionate financial burden, it is essential that structured interventions are developed and disseminated with those communities in mind. Wheeler et al. analyzed the core functions of a financial navigation (FN) intervention to address financial toxicity (FT), to facilitate its adaptation to a rural cancer context. One such structured intervention FN identifies patients at risk for or experiencing FT, educates patients about programs and services that may help address FT, directly assists patients in applying for, and receiving benefits from, existing programs and services, and tracks and manages patient needs in an ongoing manner. Their qualitative analysis provided a framework to further assess and adapt FN interventions, considering their core functions.

These studies exemplify the value of integrating dissemination and implementation lens into various levels of intervention development and assessment, as framed by the Designing for Dissemination, Implementation and Sustainability (D4DIS) models (18, 19). The D4DIS is a set of specific Fit-to-Context principles and methods for developing interventions that are closely aligned with the needs of end users and the intended context for use (19–22). Thinking about future implementation and engaging stakeholders at early stages of intervention development, recognition of the multi-level and inter-connected nature of healthcare and social systems (Figure 1), and developing strategies to facilitate coordination and alignment of different components of the implementation context are essential in implementation success and sustainment, especially with limited resources as was demonstrated by several successful learning health systems (23).
